# Induction and consolidation of calcium-based homo- and heterosynaptic potentiation and depression

**DOI:** 10.1186/1471-2202-16-S1-P252

**Published:** 2015-12-18

**Authors:** Yinyun Li, Tomas Kulvicius, Christian Tetzlaff

**Affiliations:** 1 III Institute of Physics, Department of Computational Neuroscience, Georg-August-University Göttingen, Bernstein Center for Computational Neuroscience, Göttingen, 37077, Germany

## 

Synaptic plasticity serves as the physiological foundation for learning and memory [1]. While homosynaptic plasticity is associative learning or Hebbian-type plasticity, heterosynaptic plasticity reflects the synaptic change without direct stimulation, i.e. non-associative plasticity [2]. However, heterosynaptic plasticity is an important mechanism preventing run-away synaptic dynamics and offers a potential mechanism to understand memory allocation [2,3]. Experimental results show that the induction of heterosynaptic plasticity as well as homosynaptic plasticity depends on the postsynaptic calcium concentration [4]. We propose that heterosynaptic plasticity can be induced by the postsynaptic calcium dynamics which can be triggered by the back propagation of action potentials.

However, homosynaptic plasticity has an early-phase (< 3 hours) and a late-phase state (> 8 hours) [1]. Experiments show that an early-phase synaptic change can be transferred to a late-phase by the mechanisms of "synaptic tagging and consolidation" (STC) [5,6]: (i) the changed synapse get tagged and (ii) a strong activation enables in the postsynaptic neuron the synthesis of plasticity-related proteins (PRP) which are transmitted back to the tagged synapse[5,6]. We propose that the same STC mechanism consolidating homosynaptic changes are also able to consolidate heterosynaptic changes.

We combine a history spiking-dependent neuron [7] with calcium-based synaptic plasticity rule [8] and synaptic consolidation mechanism [9] to understand: (i) the mechanisms of inducing heterosynaptic plasticity by which the inactive synapse can change its weight through the postsynaptic calcium level triggered by the back propagation of the shared neuron; and (ii) of the consolidation of heterosynaptic changes based on the synaptic tagging and consolidation principle. For instance, a strong stimulus transmitted by a group of synapses induces and consolidates by the postsynaptic neuron heterosynaptic changes at other, unrelated synapses. Our study provides a further step of understanding how several mechanisms interact with each other to enable the formation of computational important long-term changes or memories.
